# Article 10 of the Poor-Law Orders

**Published:** 1914-01-24

**Authors:** 


### THE EDITOR'S LETTER-BOX.

### Article 10 of the Poor-Law Orders.

*To the Editor of* THE HOSPITAL.

SIR,—Your very able and lucid criticism of "The New Poor-Law Orders" in your issue of January 17 prompts me to hope that you will find space for a more detailed criticism of certain Articles in those Orders. For the purpose of this letter I will restrict myself to Article 10, "Classification Within the Institution."

Sub-division 1 of this Article recites: "The Guardians shall form classes of the inmates, having regard especially to age, character, and behaviour, and shall assign to each class so formed suitable and separate accommodation," with two provisoes:

Proviso (*a*) is so patent as to require no comment; (*b*) however is in quite a different category. Let me quote that portion of it to which I would especially draw attention: "In forming classes to include . . . persons who are infirm or suffering from disease of body or mind, etc., and in assigning accommodation to those classes the Guardians shall have regard to recommendations to be obtained in writing from the medical officer." Here it will be seen that no obligation is imposed on the Guardians to comply with the recommendations of the medical officer, and the sad experience I regret to say of the majority of medical officers of workhouses is that scant attention is paid by Guardians to their recommendations unless they happen to be agreeable to them, and generally in that case for reasons entirely divorced from the welfare of the sick. An elementary knowledge of the government of workhouses might surely have suggested that there is only one officer fitted by training who should be entrusted with the duty of classifying the sick and the infirm, and that that officer is the medical officer; yet nowhere is the duty specifically imposed on him and nowhere is it imposed on the Guardians that his recommendations when made shall be adhered to. This is all the more strange when one proceeds to read paragraph (*c*), which states: "The Guardians shall comply with any directions from time to time given by the Board in regard to the classification of inmates." Might I suggest that a much more effective rendering of paragraph (*c*) would be that the Guardians shall comply with any recommendations given them by the medical officer in regard to the classification of sick and infirm inmates after such recommendations have been submitted to and approved by the Board? But no. The old bad leaven of dual control is retained, and the medical officer is retained in the old position of having responsibility without authority.

Let me now deal with Sub-division 4 of this Article. "Nothing in this Article shall affect the right of a married couple above the age of sixty to live together or the power of the Guardians to allow a married couple, one of whom is infirm, *sick*, or disabled by any injury or above the age of sixty, to live together." If this means anything, it means removing from the medical officer the power of transferring inmates from the married quarters to the sick wards for treatment without first obtaining the consent of the Guardians. It further means that the sick inmate resident in the married quarters is within his rights to refuse to obey the order of the medical officer until sanctioned by the Guardians, presumably at their next meeting. Meantime, there is impressed on the medical officer the responsibility of carrying out his professional duties under impossible conditions.

You wisely criticise the conditions by which the medical officer is subordinated to the master. This Article goes even further, for it subordinates him in addition to a lay body meeting periodically at infrequent intervals, and still further it subordinates him to a certain class of inmates of the house to which he is attached as medical officer. That, after such long delay, such an Article as Article 10 should be the outcome of the Departmental Committee's deliberations is a caustic commentary on the sources from which their information as to the government of workhouses has been derived.

For the present I sign myself

A WORKHOUSE MEDICAL OFFICER.

